# Retrograde hysterectomy approach in a patient with a frozen pelvis due to a suspected ovarian malignancy

**DOI:** 10.1136/ijgc-2022-003363

**Published:** 2022-09-28

**Authors:** Xuhui Dong, Lei Yuan, Liangqing Yao

**Affiliations:** Obstetrics and Gynecology Hospital of Fudan University, Shanghai, Shanghai, China

**Keywords:** Surgical Procedures, Operative, Laparoscopes, Hysterectomy

Surgical treatment of gynecological disease with colorectal involvement is a challenge which requires complete surgical excision of lesions.^1^ Blind blunt dissection of the Douglas pouch might cause massive and rectal tissue damage.^2 3^ Traditional hysterectomy may cause rectal injury for those cases with severe adhesion in the Douglas pouch.^4^ Retrograde hysterectomy has usually been applied in laparoscopic assisted vaginal surgery. However, are case demonstrated retrograde laparoscopic hysterectomy for a patient with severe adhesion in the Douglas pouch.

Method: A step-by-step video demonstration of the technique

Result: A 53-year-old woman with a history of dysmenorrhea had abdominal distention for 2 months. PET-CT showed that the uptake of fluorodeoxyglucose in the surrounding peripheral rim of soft tissue masses was elevated. Laparoscopy showed severe adhesions in the pelvic cavity, which might be caused by malignant tumor and endometriosis. It is difficult to expose the Douglas pouch due to the dense adhesions with poorly defined margins posteriorly to the uterus. Therefore, the surgeon decided to open the Douglas pouch finally. After coagulating the ligaments, pushing the bladder, and incising the vaginal wall, the outline and margin of the lesion was exposed so that the lesion could be cut off safely. Omentectomy, appendicectomy, partial peritonectomy, pelvic and para-aortic lymph node resection were performed. The neoplasm in the right ovarian cyst was clear cell carcinoma, and the lesion on the surface of the rectum was endometriosis. The patient recovered well without any complications and defecated 3 days after surgery. She is still alive up to now.

Conclusion: Our video showed that a flexible change in laparoscopic surgical procedures could overcome difficulty safely and quickly. Compared with the traditional type, retrograde hysterectomy can recognize the margin of a lesion, and is a safe alternative for patients with dense adhesion around the uterus.

## Data Availability

All data relevant to the study are included in the article.
